# Suicide in Recent Onset Psychosis Revisited: Significant Reduction of Suicide Rate over the Last Two Decades — A Replication Study of a Dutch Incidence Cohort

**DOI:** 10.1371/journal.pone.0129263

**Published:** 2015-06-12

**Authors:** Stynke Castelein, Edith J. Liemburg, Jill S. de Lange, Frank D. van Es, Ellen Visser, André Aleman, Richard Bruggeman, Henderikus Knegtering

**Affiliations:** 1 Rob Giel Research Center, University of Groningen, University Medical Center Groningen, Groningen, The Netherlands; 2 Lentis Research, Lentis Psychiatric Institute, Groningen, The Netherlands; 3 NeuroImaging Center, Department of Neuroscience, University of Groningen, University Medical Center Groningen, Groningen, The Netherlands; 4 University Center for Psychiatry, University of Groningen, University Medical Center Groningen, Groningen, The Netherlands; 5 Department of Psychology, University of Groningen, Groningen, The Netherlands; University of Vienna, AUSTRIA

## Abstract

This study aims to compare the suicide risk over the past decade following recent onset psychosis to findings from the eighties and nineties in the same catchment area and to identify predictors of suicide in the context of the Psychosis Recent Onset Groningen-Survey (PROGR-S). A medical file search was carried out to determine the current status of all patients admitted between 2000 and 2009. The suicide rate was compared with a study executed in 1973–1988 in the same catchment area. Predictors of suicide were investigated using Cox regression. The status of 424 of the 614 patients was known in July 2014. Suicide occurred in 2.4% of patients with psychosis disorders (n = 10; mean follow-up 5.6 years); 6 out of 10 suicides took place within two years. Within two decades, the suicide rate dropped from 11% (follow-up 15 years, 8.5% after 5 years) to 2.4%. The Standardized Mortality Rate (SMR) of suicides compared with the general population was 41.6. A higher age was the only significant predictor for suicide. Neuroticism, living situation, disorganized and negative symptoms, and passive coping style all showed a trend for significance. A significant reduction in the suicide rate was found for people with psychosis over the past decades. Given the high SMR, suicide research should be given the highest priority. Identifying predictors may contribute to further reduction of suicide among patients with psychosis.

## Introduction

Suicide remains an important cause of premature death in patients with psychotic disorders [1,2]. In early studies, the lifetime suicide risk for schizophrenia was established at approximately 10% [3,4]. This may have been caused by the conditions of patients at that time, but also with methodological approaches that may have caused overestimation of the suicide rate [5]. Indeed, more recent reviews and large studies suggest that this risk has dropped to 5% [5–7]. Dutta et al. even found lower suicide rates of 1.9% (follow-up 11.5 years) [8]. Similarly, up to 60% of patients with schizophrenia reported suicidal ideation and/or attempted suicides in studies published a decade ago [9,10]. Albeit some studies still find prevalence of approximately 60% of suicidal ideation [11] and methodological progress may have caused differences in findings, other recent studies show reduced rates, with a prevalence of approximately 20% [12,13]. Whether such a decrease in suicide risk and suicidal behavior can be replicated and also holds for the Netherlands remains to be determined.

Numerous studies have been carried out with the aim of identifying predictors of suicide risk in patients with psychotic disorders. As suicide risk peaks in the first years after onset, much attention has been given to this phase of the illness [5]. It has been argued that early intervention is important in preventing suicide [14,15], although not all studies showed an effect [16,17]. The aim of the current study is to investigate the risk factors of suicide in recent onset psychosis. In the following paragraphs, relevant literature concerning the investigated risk factors will be described briefly.

The relation between suicide risk and age of onset of psychotic symptoms is complex. Some studies have reported a higher risk in patients with earlier age of onset [18,19], others have found a relationship with later onset [20–23] and some failed to find any relationship at all [16,17]. With regard to gender, males seem to have a greater tendency to commit suicide [1,24–26], although the opposite has also been reported, with female patients having a higher risk of suicide and suicidal behavior [18,27].

There are no consistent findings on the influence of social status on suicide risk. Whereas one study showed a trend that cohabiting decreases suicide risk [28], a more recent study found a non-significant association in the opposite direction [22,29]. With regard to employment, being unemployed did not impact suicide risk [22] or may even decrease the risk [29,30].

Suicide risk has also been reported in association with higher education [22,29,31]. The same holds for intelligence: the higher the IQ, the higher the suicide risk [32,33], although not all studies found an association between IQ and suicide risk [10,19].

There is conflicting evidence on the predictive value of specific symptom domains on suicide risk. Thus, severity of positive symptoms was found to be associated with a higher suicide risk [19], but more recent studies did not show any effect [16,17]. Interestingly, in 1985, Drake et al. demonstrated that patients committed suicide in a relatively non-psychotic phase of the illness [31]. A significant lower long-term risk for suicide was found when negative symptoms were present as a prominent component of illness in patients with schizophrenia spectrum disorders [9,34]. Moreover, Doihara et al. and Wu et al. have suggested that suicide attempters had higher impulsivity [35,36]. There is strong evidence for depressive disorders or elevated levels of depressive symptoms as a significant risk factor for suicide [28,30,37–39]. Indeed, depressed mood is often reported by attempters as one of the reasons for suicidal behavior [14,33,40].

Little is known about the relationship between personality traits, coping styles and suicide risk in schizophrenia. In the general population, higher levels of neuroticism may increase the risk for suicide [41]. In addition, higher levels of neuroticism seem to predict future onset of psychotic disorders [41,42]. A large study by Li and Zang showed that patients with suicidal ideations, both with and without psychotic disorders, scored significantly higher on passive coping mechanisms and lower on active coping than the control group [43]. However, studies on neuroticism and coping styles in relation to suicide risk in psychotic patients are lacking.

### Aims of the study

The primary aim of the present study was to investigate the change in suicide risk in recent onset psychosis by replicating a study on suicide risk two decades later in the same catchment area (cf. Wiersma et al. 1998). Based on innovations in mental health care over the past two decades, we hypothesize that suicide risk will be lower compared with two decades ago. Second, we aim to investigate the possible predictors of suicide risk, which may give indications for effective treatment options to prevent suicide. We expect that suicide risk will be higher in patients with male gender, older age, living alone, being unemployed, higher education level, higher IQ, longer presence of positive symptoms, depressive symptoms, neuroticism and a passive coping style. Protective factors could be female gender, the presence of negative symptoms and an active coping style.

## Materials and Methods

### Study sample

The diagnostic protocol of the Psychosis Recent Onset Groningen—Survey (PROGR-S) started in 1997 for those living in of the province of Groningen in the north of the Netherlands with 550,000 inhabitants, who were referred to a psychiatric institute with a suspected first psychotic episode (< 2 years) or were evaluated for a recurrent psychotic episode not diagnosed as such before. There were no exclusion criteria with regard to age, diagnoses, substance abuse, or ethnicity. All data were collected for clinical purposes and after the process had been fully explained, participants gave oral and written informed consent to the use of their data for research purposes. Only persons eligible to provide informed consent were included, i.e. they should understand their current condition, understand the information provided and respond accordingly. A final informed consent was obtained after the protocol was completed (6 – 8 weeks) and florid psychotic symptoms had ceased. All procedures were in accordance with local and international rules as confirmed by the local ethical committee of the University Medical Center of Groningen (Medische Ethische Toetsingscommissie UMCG). The medical ethical committee of the University Medical Center Groningen declared that their approval was not required, as data were collected for diagnostic purposes, no interventions outside standard care were performed, and data were anonymized for research purposes. In total, 614 patients were included (2000–2009 period). An extensive description of the survey can be found elsewhere [44].

### Data collection

Database and patient file search were used to identify cases where patients had committed suicide. File search was conducted by JSL, assistant physician member of the treatment team, under supervision of RB and HK, both psychiatrists and heads of the involved departments. An anonymous, coded file was provided to the researchers SC and EJL. Beforehand, this procedure was discussed with an external data protection committee that approved that procedures were conducted in accordance with the Dutch law.

First, the clinical data were connected to the Northern Netherlands Psychiatric Case Registry (PCRNN), which monitors all health care consumption by individuals attending mental health care organizations from the northern Netherlands on a daily basis. For most subjects, data could be linked to the PCRNN, showing the institute where the last mental health care consumption was registered. Based on this information, a file search could be carried out at these institutions to determine the status of the subjects as of 1 July 2011. Of the subjects for whom PCRNN data were not readily available, an additional file search was carried out across all databases. We determined whether a subject had committed suicide, or if he/she was still in care, moved out of the catchment area, or had died due to causes other than suicide up to July 2011. In some cases, the current status of a subject could not be ascertained from the file search. The status of these subjects was classified as ‘unknown’. The patients of whom the status was unknown at the end of the study period, were not censored, because a specific end point could not be reliably determined and may differ substantially between patients.

### Measures

Demographic characteristics were reported for reference. These included age, gender, highest completed level of education, occupation, and living situation. Clinical characteristics included symptom-severity as assessed by the Positive and Negative Syndrome Scale (PANSS) [45], Depression was measured with the Montgomery-Asberg Depression Rating Scale (MADRS) [46]. The use of antipsychotics was noted and expressed as haloperidol equivalents [47]. The DSM-IV diagnosis of patients was based on the Schedules for Clinical Assessment in Neuropsychiatry (SCAN) [48]. First-episode psychosis was established by interrogating lifetime psychotic experiences. The eventual diagnosis was based on all information collected in the PROGR-S protocol and consensus was reached in a multidisciplinary meeting with SCAN-trained psychiatrists, psychologists and nurse practitioners. IQ was calculated based on outcome of the Wechsler Adult Intelligence Scale (WAIS-III) [49]. Personality traits were measured based on the Neuroticism-Extroversion-Openness Five Factor Inventory (NEO-FFI), which is a self-report questionnaire that measures the Big Five personality traits [50]. Coping strategies were assessed using the Utrecht Coping List (UCL) [51].

The hypothesized variables were prepared for the subsequent Cox regression survival analysis. Categorical variables were grouped into few categories to save degrees of freedom in the Cox regression. The following demographic data were included: Age, gender (male/female), IQ, occupation defined as ‘yes’ (paid or voluntary job, student or running a household) or ‘no’ (unemployed or disabled), living together (married, living with parent(s) or living with family/partner) divided into ‘yes’ or ‘no’ (living alone, at a mental health institute or homeless). Positive, negative, excited, and disorganized symptoms were assessed with the PANSS [52]—the depression dimension was excluded. Depression was measured with the (MADRS), from which the total score and the score on the suicide item were drawn. Neuroticism was extracted from the NEO-FFI. Active and passive coping styles were derived from the UCL. Due to the number of missing values, duration of Illness (DUP) was not included in the analysis.

### Statistical analysis

Statistical analyses were performed using the SPSS statistical package version 20.0 for Windows (IBM SPSS Statistics 20). The main demographic and clinical variables were compared between patients for whom the situation (in care, out of care, deceased or moved) was identified at the 1st of July 2011 (the ‘known’ group) and patients for whom their status not known at that date (the ‘unknown’ group). The Shapiro-Wilk test was conducted to test for normality. Mann-Whitney U tests were used to compare both groups on continuous variables, because data were non-normally distributed, and Chi-Square tests for independence were used for the categorical variables.

First, the risk of suicide over the years was determined after the PROGR-S diagnostic protocol, without the effect of covariates. An event was defined as completed suicide. Data were censored for subjects who were lost to follow up as a result of moving elsewhere, death or leaving psychiatric care. Kaplan Meier regression with a log rank test was used to compare the suicide rate of the current sample with the sample collected by Wiersma et al. [4]. To test whether there was an effect of differences in demographic variables in both samples, age in six categories, gender, living situation, and occupation were added as strata in this regression. The standardized mortality rate (SMR) comparing the suicide rate in our cohort with the general Dutch population was also calculated. Mortality rates were drawn from Statistics Netherlands data (www.cbs.nl) and were categorized into age groups (< 15 years, 15 – 20, 20 – 30, …, > 80), gender, and year of occurrence (2000 – 2011). The expected number of suicides for each group was calculated and then the SMR was calculated as follows: (expected suicides/100,000/year) / (observed suicides/100,000/year). The significance was determined by calculating the 95% confidence intervals.

Next, Cox regression was performed with all aforementioned variables in the model. Covariates were stepwise removed from the model using the Likelihood Ratio test with p < 0.1 to select the optimal model.

Because the number of suicide events was limited and some cases had missing values for IQ and the UCL and NEO, multiple imputations were used for the remaining variables in the model. Continuous variables were estimated with linear regression and categorical variables (gender, occupation, and living situation) with logistic regression. The range of variables was restricted to realistic values. Age was restricted from 16 to 120 years and questionnaire and interview subscales to their minimum and maximum scores. Fifty imputed datasets were generated. After imputation, averages, standard deviations and distributions were compared between the original and imputed data for continuous variables, and ratios were checked for categorical variables. Cox regression analysis was applied to the imputed dataset without further elimination of covariates. This implies that Cox regression was performed 50 times on every imputed dataset separately, and then combined for pooled statistics.

## Results

**Fig 1:**
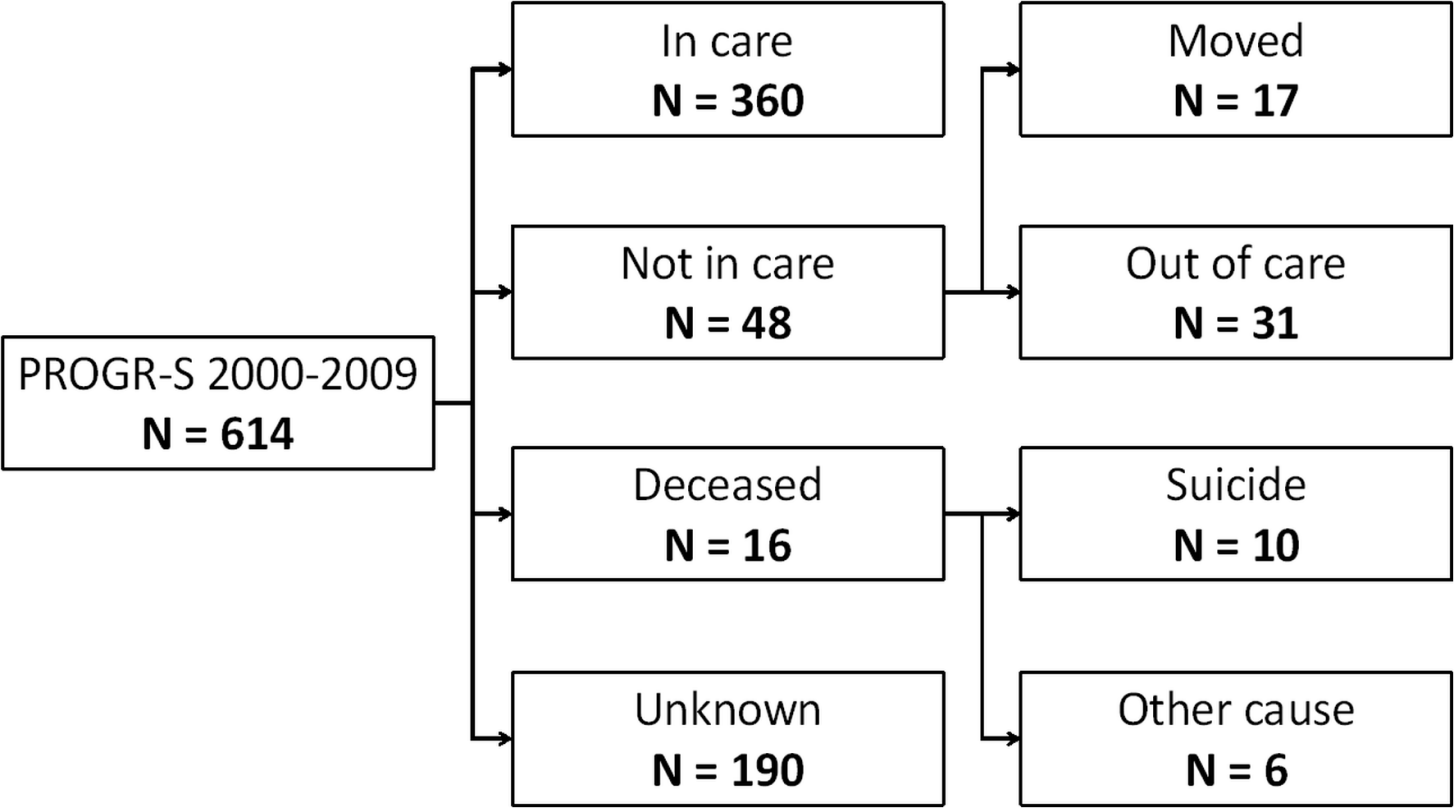
Flow diagram of patient status at July 2011, the unknown group was lost to follow up and not included in analysis.

Of the 614 individuals in the PROGR-S database, the status of 424 was known as of July 1st 2011, as shown in Fig 1. For dataset see S1 Dataset. For 190 subjects, there was no recent correspondence in the mental health care medical file system. This group of subjects was lost at follow-up and classified as ‘unknown’. An overview of the demographic data on both groups is presented in S1 Table. The unknown group had a significantly lower age (27.4 years vs. 28.5 years) and less positive symptoms (6.7 vs. 7.7). Age and positive symptoms were significantly different between the known and unknown groups. The demographic characteristics of Wiersma et al. are also shown for reference [4].

**Fig 2:**
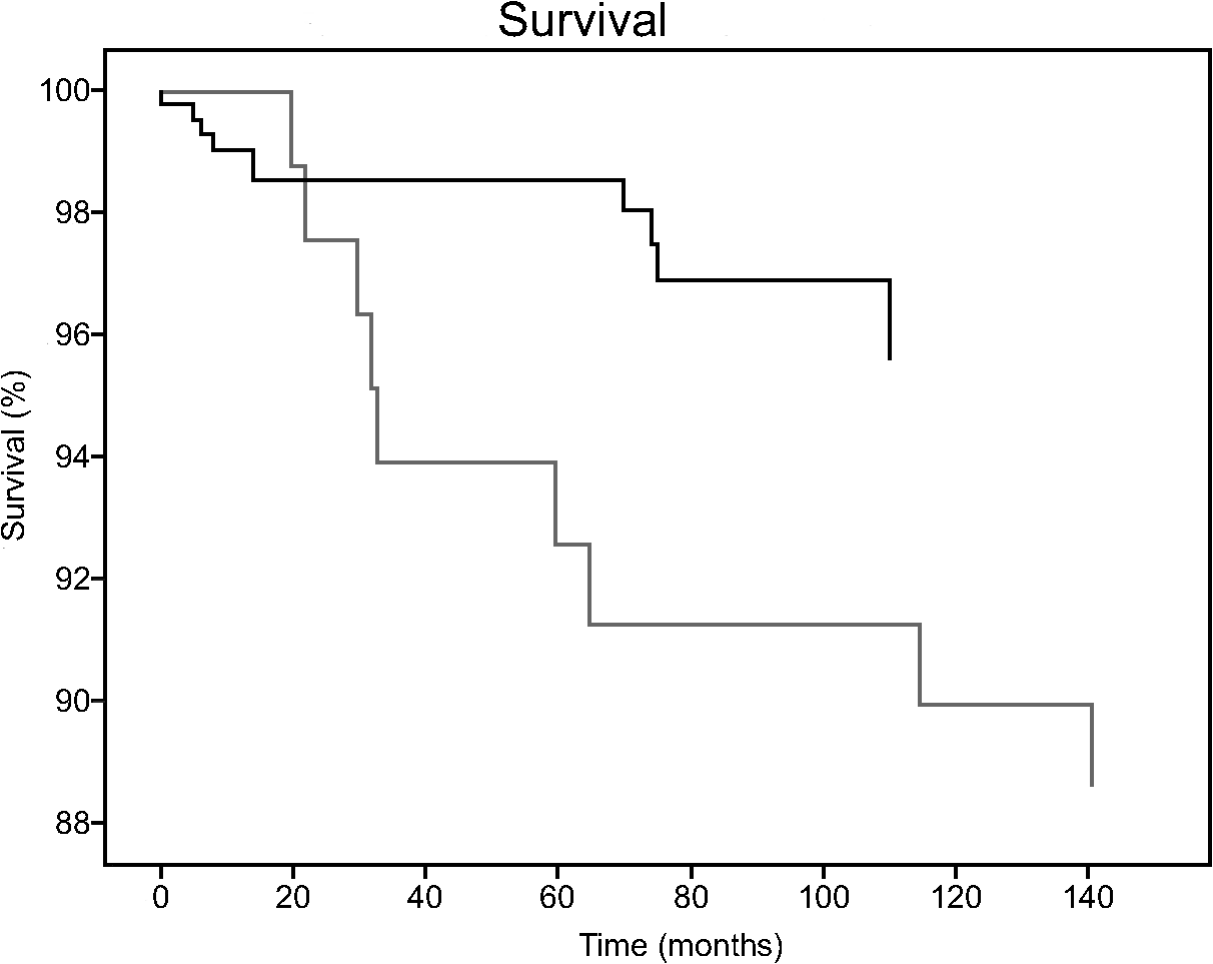
Survival curves of both samples, the known PROGR-S sample (n = 424; black) censored for patients that were out of care at a certain point in time; a graph based on Wiersma et al., 1998 [3] (n = 85; gray) is shown for referential purposes.

Ten individuals had committed suicide before July 1st 2011, and six had died of other causes. This implies that 2.4% of the ‘known’ subjects committed suicide within an average follow-up of 5.6 years (range: 1 week-11.6 years). The cumulative suicide rate, which takes dropouts into account, was 4.3%. Six suicides (1.4% of the suicides) occurred within the first two years, with four in the first year. The other suicides occurred 6, 7 and 9 years after the PROGR-S measurement. The survival graph is shown in Fig 2. The graph is corrected for the number of known cases in care at that point in time. The survival curve plotted by Wiersma et al. [4] displaying the 1973–1988 period is also shown. The log rank test showed that there was a significant difference in the survival rate (Chi^2^ = 5.3, p = 0.021). Results remained significant after adding age (Chi^2^ = 7.3, p = 0.007), gender (Chi^2^ = 5.7, p = 0.019), and living situation (Chi^2^ = 9.4, p = 0.002) as stratum. Occupation could not be tested, because there were no cases in the Wiersma sample without occupation.

Causes of death, other than suicide, included herpes simplex encephalitis, acute lymphoblastic leukemia, malignancy, and, presumably, Wernicke encephalopathy. The remaining causes of death were unknown. The SMR for suicides compared with the general population was 41.62 (4162%); 95% CI = [37.67 – 45.6].

Analysis of patterns of missing data showed that data were randomly missing across cases. After imputation, the mean and distribution of continuous variables in the imputed sets was highly similar to the original sets, as were ratios for categorical variables. Averages, standard deviations, and ratios for the predictors in the model from the original dataset are given in Table 1. Cox regression was performed on the original dataset and the imputed dataset. Results of the original dataset are shown in Table 2. All data were available for 7 suicide cases and 297 other cases. The analysis without imputation showed a significant effect for age, neuroticism and passive coping, and a trend for significance of living situation, negative, and disorganized symptoms.

**Table 1:** Overview of potential predictors of suicide for the suicide group and the other patients from the original dataset.

	Suicide		Others	
	Mean/%	SD	Mean/%	SD
Age	35.8	9.2	28.3	8.9
Gender (% male)	70.0		71.3	
Living with others (%)	20.0		45.2	
Having occupation (%)	60.0		54.3	
IQ	109.4	27.5	96.4	17.7
Positive symptoms	7.7	2.5	7.8	3.6
Negative symptoms	9.6	3.7	13.0	5.7
Disorganized symptoms	5.1	2.7	5.5	2.7
Excited symptoms	4.9	1.3	5.4	2.1
Depression	10.7	9.2	13.1	8.8
Suicidal thoughts	0.9	1.5	0.8	1.2
Neuroticism	33.8	7.8	37.7	7.9
Active coping	19.3	3.0	16.9	3.7
Passive coping	14.9	4.2	15.2	4.2

**Table 2:** Results of Cox regression analysis of complete cases in original dataset (n = 7).

	B	SE	Sig.	Exp(B)	95% CI for Exp(B)	
					Lower	Upper
Age	0.2	0.1	0.003*	1.2	1.1	1.3
Living with others	-2.3	1.2	0.057	0.1	0.009	1.1
Negative symptoms	-0.2	0.1	0.054	0.8	0.6	1.0
Disorganized symptoms	0.4	0.2	0.051	1.5	1.0	2.3
Excited symptoms	-0.9	0.5	0.116	0.4	0.1	1.2
Neuroticism	-0.3	0.1	0.007*	0.8	0.6	0.9
Passive coping	0.5	0.2	0.036*	1.6	1.0	2.5

^* Significant association^

When the analysis was repeated with these covariates in the imputed dataset, only age remained significant and neuroticism showed a trend. Pooled results are shown in S2 Table.

## Discussion

This study demonstrated a suicide risk of 2.4% during a mean follow-up period of 5.6 years in individuals with recent onset psychosis in the Northern Netherlands (period: 2000 – 2009), which is significantly lower than two decades earlier (1973 – 1988). The suicide risk was highest in the first two years following the onset of psychosis. Later age of onset was the only strong predictor of suicide confirmed in the imputed dataset. Neuroticism was a moderate predictor showing a trend toward significance in the imputed dataset. Living situation, negative and disorganized symptoms, and passive coping may also predict the risk of suicide, although the findings are less convincing for these variables.

The finding of a decline in suicide rate from 11.0% (follow-up at 15 years; 8.5% after 5 years) to 2.4% (mean follow-up 5.6 years, cumulative rate 4.3%) within two decades was not affected by differences in age, gender, and living situation between both samples. We could not investigate the effect of occupation, but this was not a significant predictor of suicide in our recent sample. Our finding is consistent with a large naturalistic study [53]. Moreover, these data confirm a recent Western European study showing a suicide rate of 1.9% (mean follow-up of 11.5 years) with most of the suicides occurring within the first two years [8]. A recent large worldwide study also showed a low incidence [54]. A meta-analysis in 2005 found an intermediate suicide rate of 4.9% in the intervening period, supporting the gradual decline over the years [5].

Although we can only speculate, improvements in mental health care may have had a major impact on suicide risk. In the late 1970s and 1980s, psychiatric care delivered to patients with psychotic disorders in the Netherlands was characterized by inconsistent, less adequate pharmacotherapy and a lack of rehabilitation focusing on daily living, work as well as low levels of independence for more severe patients [4]. Nowadays, in the Groningen-area, all patients with recent onset psychosis are referred for a standardized diagnostic procedure with validated instruments; and all clinical data are collected in the Psychosis Recent Onset in Groningen Survey (PROGR-S) [44]. On the basis of multidisciplinary guidelines, evidence-based interventions are offered as part of an Integrated Psychosis Care Program, including: pharmacotherapy (relatively low dosages, majority second generation antipsychotics), cognitive behavioral therapy, an active rehabilitation and recovery oriented approach (e.g. Individual Placement and Support) [55]. The program also includes a focus on the patient’s perspective e.g. on side effects [56], extensive psycho-education, and annual Routine Outcome Monitoring (ROM) of all patients with psychotic disorders. It has been shown that patients in communities with an early detection program have a significantly lower rate of suicidal behavior compared with others [57].

While the decline of suicide is substantial in the study group, the mortality rate due to suicide is still alarming. Thus, patients diagnosed with recent onset psychosis have a markedly increased chance of completing suicide, as the reported deaths were 4162% the expected rate in the general population. This underlines the importance of studying the predictors of suicide [58].

We found a higher age during assessment to be the only independent risk factor for suicide. With each year, the risk of suicide increased by 1.1%. This finding corresponds with earlier results [20–23]. Individuals assessed later in life, the majority of whom experienced a later onset of psychosis, might find it more difficult to accept the changes and limitations associated with the diagnosis, as many have built a family, career and future plans. While age is a fixed factor, therapy could focus more on illness related losses that patients experience. Moreover, we observed a trend for living situation; only in 20% of the suicide cases, the individual concerned lived together with another person, as opposed to 45% of the other cases, suggesting that living together may be a protecting factor. Previous studies on this subject showed varying results. Whereas one study showed a similar trend [28], others found a trend in the opposite direction [22,29]. Other socio-demographic characteristics, *i*.*e*. gender and occupation, showed no relation with suicide risk. Studies that showed a higher risk in males had larger samples, a longer follow-up or more females in the sample [1,24,25]. With regard to occupation, there was no influence on the suicide risk as demonstrated previously [28].

Most studies report that higher education or a higher IQ is associated with an increased risk for suicide [22,31,33], and only a few studies support our finding that intelligence had no effect [10,19]. Of note: we observed that the IQ of suicide cases was on average almost 15 points higher than other cases; however, the high standard deviation may have caused a lack of power.

As expected, more negative symptoms showed a trend for significance in relation to a decreased suicide risk. It has been shown that negative symptoms may protect against suicide risk [9,34]. A similar mechanism may underlie the finding that fewer disorganized symptoms correlate with a higher suicide risk. There was no effect for positive and excited symptoms, in line with some previous findings for positive symptoms [16,17], although other studies did show a significant association [19,23].

Notably, no clinically relevant difference was observed between groups on suicidal ideation or depression. Studies in which depression was reported as a risk factor for suicide measured the degree of depression prior to suicide [37,38], although meta-analyses are able to detect an effect of prior depression [28,30]. In our results, we observed more suicidal ideation and less severe negative symptoms in patients who committed suicide within two years after PROGR-S compared with suicide cases after two years and non-suicide cases. Thus, clinicians should be aware that diagnostic screening on symptoms might predict suicide in the short-term, but not the long-term. Fleischhacker et al. showed that monitoring the history of suicide attempts might help to predict increased suicide risk [54]. Indeed, half of the cases in our sample had previous suicide attempts.

Patients who completed suicide within two years referral had lower levels of neuroticism and showed more passive coping, suggesting that neuroticism may be predictive in the short term for suicide risk. While our findings fit our hypothesis for passive coping [59], we expected that higher levels of neuroticism would be related to suicide risk [41,42]. One could speculate that higher levels of neuroticism and passive coping may also protect against suicide in a similar way to negative symptoms. There was no effect for an active coping style.

The major strength of the present study was the broader inclusion criteria of different diagnoses compared with most studies, since all psychotic diagnoses were incorporated. Another strength is that this study had the same catchment area as the study of Wiersma et al. [3] enabling us to compare suicide rates over time.

A limitation of this study was that data on a part of the patients was lost to follow-up. The patients of whom the status was unknown at the end of the study period were not censored because a specific end point could not be reliably determined and may differ substantially between patients. A patient may have moved, deceased, refused further care, etcetera. Addition of those patients may bias the suicide rate to even lower values. It is possible, that suicide rates are higher in the group lost to follow-up, e.g. suicidal persons may avoid contact with mental health services [60]. However, we do not expect that this influenced the findings, as the ‘known’ and ‘unknown’ groups did not differ on most characteristics. Unfortunately, data on symptom severity was not available in the Wiersma sample, as was detailed data on the diagnostic procedure. Moreover, DUP of the current sample could not be rigorously evaluated sample had a high diversity in diagnoses due to the naturalistic setting, while suicide may differ between diagnoses [7]. It would be interesting to investigate DUP, along with previous attempts and hospitalizations in a larger sample, so factors like diagnosis can be taken into account [54]. It would also be desirable to have follow-up data on interviews, because we were not able to investigate whether current symptom state predicted the suicide risk. Finally, the low prevalence of suicide, which in itself is positive, combined with missing values, may have caused lack of statistical power in the determination of all predictors of suicide. However, our finding of a significant effect of age had a power of 0.6, which is moderate. Finally, missing values are inevitable in the clinical setup of PROGR-S. In the future, we plan to repeat the analysis with a larger sample.

## Conclusion

To conclude, a considerable drop in suicide rate was found over the past two decades in the north of the Netherlands in patients with psychotic disorders. Nonetheless, research and prevention of suicide in psychosis should have the highest priority, as the risk is still very high when compared with the general population.

## Supporting Information

S1 Table Demographic data of subjects whose status was known at the end of the measurement period (n = 424) and subjects whose status was unknown (n = 190). The last column contains demographic information of Wiersma et al. (1998) for comparison.
(DOCX)

S2 Table Pooled results of Cox regression analysis of imputed dataset (n = 10). (DOCX)

S1 Dataset Dataset used for the survival analyses. Both recent dataset and the dataset of Wiersma et al. (1998) are included.
(ZIP)
