# Supplementary material for: Suicide in Recent Onset Psychosis Revisited: Significant Reduction of Suicide Rate over the Last Two Decades — A Replication Study of a Dutch Incidence Cohort
Source: PLoS One. 2015 Jun 12;10(6):e0129263. doi: 10.1371/journal.pone.0129263 (PMC4466318; doi:10.1371/journal.pone.0129263)
Supplement: S1 Table — The last column contains demographic information of Wiersma et al. (1998) for comparison. (DOCX) [file pone.0129263.s001.docx]

**Supporting information**

Table S1. Demographic data of subjects of whose status was known at the end of the measurement period (n = 424) and subjects whose status was unknown (n = 190). The last column contains demographic information of Wiersma et al. (1998) for comparison

|  | Known |  | Unknown |  |  | Wiersma et al. |
| --- | --- | --- | --- | --- | --- | --- |
|  | Mean/% | SD | Mean/% | SD | P-value |  |
| Age | 28.5 | 9.0 | 27.4 | 9.2 | 0.049 | 24.4^1^ |
| Gender (% male) | 71.2 |  | 78.4 |  | 0.077 | 52.4 |
| Living situation |  |  |  |  | 0.35 |  |
| *Alone* | 48.6 |  | 54.2 |  |  | 17.1 |
| *Parent(s)* | 31.8 |  | 31.1 |  |  | 37.8 |
| *Partner/family* | 12.8 |  | 11.1 |  |  | 32.9 |
| *Mental health institute* | 3.8 |  | 1.6 |  |  | 5.0^2^ |
| *Homeless* | 1.7 |  | 0.5 |  |  | - |
| Occupation (%) |  |  |  |  | 0.37 |  |
| *Unemployed* | 40.3 |  | 43.7 |  |  |  |
| *Paid job* | 33.0 |  | 32.1 |  |  | 19.5 |
| *Voluntary job* | 5.2 |  | 7.4 |  |  | - |
| *Student* | 15.6 |  | 11.6 |  |  | 43.9 |
| *Running household* | 0.7 |  | 0 |  |  | 36.6 |
| Highest completed level of education (%) |  |  |  |  | 0.073 |  |
| *Secondary school* | 15.3 |  | 22.6 |  |  | 34.2 |
| *High school* | 7.8 |  | 9.5 |  |  | - |
| *Vocational education* | 33.7 |  | 31.6 |  |  | 65.9 |
| *College* | 39.2 |  | 31.1 |  |  | - |
| IQ | 96.7 | 18.0 | 96.6 | 18.8 | 0.83 | - |
| Diagnosis (%) |  |  |  |  | 0.11 |  |
| *Schizophrenia* | 45.3 |  | 36.3 |  |  | 46.0 |
| *Substance induced psychosis* | 4.5 |  | 4.2 |  |  | - |
| *Psychotic disorder* | 19.1 |  | 23.2 |  |  | 3.2 |
| *Schizoaffective disorder* | 5.7 |  | 2.6 |  |  | - |
| *Delusional disorder* | 3.5 |  | 3.2 |  |  | - |
| *Bipolar disorder* | 4.2 |  | 7.4 |  |  | 7.9 |
| *Affective disorders* | 5.9 |  | 5.8 |  |  | - |
| *Other diagnosis* | 3.1 |  | 6.8 |  |  | 42,9^3^ |
| Positive symptoms | 7.7 | 3.7 | 6.7 | 3.9 | <0.0005 |  |
| Negative symptoms | 12.8 | 5.7 | 12.7 | 6.2 | 0.65 |  |
| Disorganized symptoms | 5.5 | 2.7 | 5.7 | 3.1 | 0.52 |  |
| Excited symptoms | 5.3 | 2.1 | 5.5 | 2.7 | 0.81 |  |
| Depressive symptoms | 7.5 | 3.1 | 7.5 | 3.0 | 0.56 |  |
| Antipsychotics (%) |  |  |  |  | 0.24 |  |
| *Medication naïve* | 17.9 |  | 23.7 |  |  |  |
| *Oral monotherapy* | 71.7 |  | 66.3 |  |  |  |
| *Polypharmacy/depot* | 8.7 |  | 7.9 |  |  |  |
| Haloperidol equivalents^4^ | 6.3 | 3.9 | 6.2 | 3.9 | 0.80 |  |

^1 Average estimate based on age categories^

^2 Higher inpatient care reflects care in that period^

^3 No detailed information on diagnosis^

^4 Based on Andreasen NC, Pressler M, Nopoulos P, Miller D, Ho BC. (2010) Antipsychotic dose equivalents and dose-years: A standardized method for comparing exposure to different drugs. Biol Psychiatry 67: 255-262.^

s
