# Supplementary material for: Suicide in Recent Onset Psychosis Revisited: Significant Reduction of Suicide Rate over the Last Two Decades — A Replication Study of a Dutch Incidence Cohort
Source: PLoS One. 2015 Jun 12;10(6):e0129263. doi: 10.1371/journal.pone.0129263 (PMC4466318; doi:10.1371/journal.pone.0129263)
Supplement: S2 Table — (DOCX) [file pone.0129263.s002.docx]

Table S2. Pooled results of Cox regression analysis of imputed dataset (n = 10)

|  | **B** | **SE** | **Sig.** | **Exp(B)** | **95,0% CI for Exp(B)** | |
| --- | --- | --- | --- | --- | --- | --- |
|  |  |  |  |  | **Lower** | **Upper** |
| Age | 0.109 | 0.4 | 0.003* | 1.1 | 1.0 | 1.2 |
| Living with others | -1.204 | 0.8 | 0.14 | 0.3 | 0.06 | 1.5 |
| Negative symptoms | -0.151 | 0.09 | 0.076 | 0.9 | 0.7 | 1,0 |
| Disorganized symptoms | 0.082 | 0.1 | 0.54 | 1.1 | 0.8 | 1.4 |
| Excited symptoms | -0.168 | 0.2 | 0.49 | 0.8 | 0.5 | 1.4 |
| Neuroticism | -0.125 | 0.07 | 0.059 | 0.9 | 0.8 | 1.0 |
| Passive coping | 0.163 | 0.1 | 0.25 | 1.2 | 0.9 | 1.6 |

^* Significant association^
